# A Call to Recognize the Integral Role of Physician Associates and Nurse Practitioners in Modern Health Care: Editorial

**DOI:** 10.2196/89262

**Published:** 2026-01-23

**Authors:** Amy Price, Kathleen Price

**Affiliations:** 1 Journal of Participatory Medicine, JMIR Publications, 130 Queens Quay East, Unit 1100, Toronto, ON, M5A 0P6, Canada, 1 561 843 7372; 2 Colorado School of Public Health, Anschutz, CO, United States; 3 St. Thomas University, Miami, FL, United States

**Keywords:** nurse practitioner, nurse anesthetist, physician assistant, participatory medicine, equity, evidence-based policy, patient safety

## Abstract

Policies governing health care professionals must be evidence-informed and include meaningful representation of all stakeholders, or commitments to quality and equity will remain shallow rhetoric. Physician associates (PAs), nurse practitioners (NPs), and patients deserve full participation in decisions affecting practice and patient care. The current health care landscape faces unprecedented workforce challenges, requiring a shift toward evidence-informed policy and the meaningful representation of all stakeholders. This editorial aims to advocate for the full participation of PAs, NPs, or advanced practice providers and patient representatives in clinical and policy decisions, contrasting established global models with emerging frameworks to promote a more practical, team-based hierarchy. While recent reviews in the United Kingdom highlight a lack of localized, high-quality data, extensive evidence from the United States and other international contexts demonstrates that PAs and NPs provide safe, effective care with clinical outcomes comparable to physicians. We argue that recognizing these professionals as integral members of the health care workforce, rather than mere stopgaps, is essential for improving care quality and patient well-being. This editorial recommends standardized credentialing, integrated educational pathways, and the inclusion of patient representatives as voting members in policy decisions to foster a truly participatory medicine model.

## Introduction

We appreciate the thoughtful, emergent review by Greenhalgh and McKee [1], which provides timely insights into the evolving roles of physician associates (PAs) in the UK health care system. The authors’ rapid scoping review, while acknowledging the limited and variable-quality data, emphasizes the need for robust evidence to inform policy and practice. We share their commitment to high-quality research and believe it is time to honor the significant contributions of PAs and nurse practitioners (NPs) as advanced practice providers, who are integral as professionals in health care, with equal access to recognized professional designations to include educational and research funding, especially given the substantial evidence of benefit from the United States.

## Understanding the Roles of PAs and NPs as Advanced Practice Providers

It is critical we understand that in other countries, training and roles vary. For example, in the United States, NPs are advanced practice registered nurses who complete graduate-level education (master’s or doctoral) and are nationally certified in their specialty areas. They practice independently or collaboratively, depending on state regulations, while providing a wide range of health care services in primary, acute, and specialty care settings [2]. PAs in the United States undergo rigorous medical education, including obtaining a master’s degree and performing clinical rotations, equipping them to diagnose, treat, and manage patient care collaboratively with physicians, delivering care in primary, emergency, and specialty settings.

In the United Kingdom, PAs undergo a shorter training pathway, typically including a first degree and 2 years of postgraduate education, and work only under physician supervision. Unlike in the United States, PAs in the United Kingdom are not yet authorized to prescribe medications or order ionizing radiation independently [1]. The United Kingdom’s PA/NP model is still evolving, and comparisons with the more established US model can provide valuable insights into optimizing their roles.

## Evidence of Safety, Efficacy, and Value

Contrary to perceptions of limited evidence, global data demonstrate that advanced practice providers contribute significantly to health care systems, particularly in underserved and rural areas. These professionals provide safe, effective care, with clinical outcomes comparable to those of physicians [3]. Quantitative evidence from the United States and Canada further supports their value:

- Safety and malpractice—advanced practice providers are associated with lower rates of safety incidents and malpractice claims compared to traditional models, suggesting a high standard of patient safety and adherence to scope of practice [4].
- Cost and efficiency—meta-analyses of randomized controlled trials indicate that advanced practice providers positively impact health care costs while maintaining or improving quality of care and patient well-being [5-8].
- Primary care performance—in primary care environments, advanced practice providers consistently manage patient volumes equivalent to physicians while maintaining high patient satisfaction and positive health outcomes [8,9].
- Economic impact—systematic reviews of economic evaluations confirm that incorporating advanced practice providers can reduce overall health care expenditures while maintaining high-quality outcomes [9,10].

Furthermore, advanced practice providers demonstrated remarkable adaptability during the COVID-19 pandemic, filling critical gaps and maintaining care continuity during extreme system stress. Responsible advanced practice providers carefully work within their scope of practice to protect the best interests of their patients and reduce institutional liability.

## Addressing Concerns With Collaboration and Clarity

**Figure 1.**
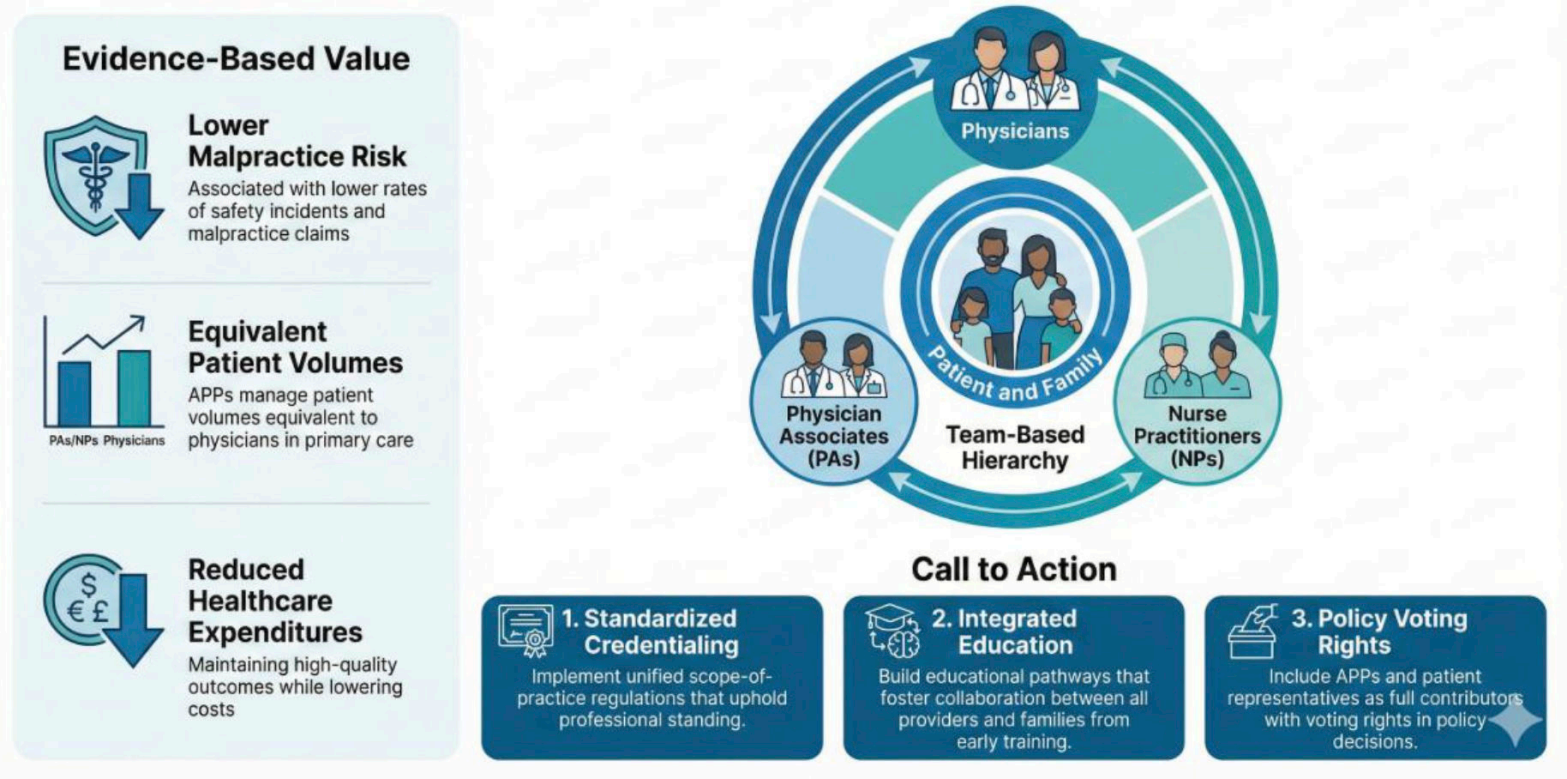
The integrated care model: beyond the stopgap. APP: advanced practice provider.

Concerns about supervision and accountability, as highlighted in Greenhalgh and McKee’s review [1], underscore the need for clear scopes of practice and well-defined roles within health care teams. In the United States, supervisory and collaborative agreements between PAs, NPs, other advanced practice providers, and physicians are governed by state laws and institutional policies, providing structured frameworks for safe and effective practice. Such frameworks could serve as models for the United Kingdom and in other areas where a national scope of practice for advanced practice providers is still under development. We draw the attention of readers to the integrated care model shown in Figure 1.

The collaborative nature of advanced practice providers enhances, rather than burdens, physician workflows. By managing lower-acuity cases and supporting team-based care, they allow physicians to focus on more complex cases, ultimately improving care delivery and reducing burnout among all health care providers. Recent research highlights how advanced practice providers play an essential role in sustaining health care systems amid workforce shortages, particularly in rural and underserved areas; worldwide, we face acute shortages of medical providers, particularly in rural and low-income areas [10].

## Conclusion: A Path Forward With Evidence and Appreciation

As health care systems worldwide grapple with workforce challenges and rising costs, it is crucial to acknowledge and support the roles of advanced care providers, inclusive of PAs and NPs. We recommend a call to action:

First, develop standardized credentialing and scope-of-practice regulations that recognize and uphold advanced practice provider credentialing in state, federal, and international policy and provide advanced care providers with the same access to funding and principal investigator status as other medical professionals. Second, ensure recognition and respect for advanced practice providers. While patients and families anecdotally praise their advanced practice providers for compassionate care and sensitivity in end-of-life discussions, informed shared decision-making, and coordination with other services, this is notably absent in the literature. Third, create integrated educational pathways that foster collaboration between physicians, PAs, NPs, patients, and their families from early training stages. Fourth, implement supportive supervision models that balance autonomy with appropriate oversight. Fifth, include advanced practice providers and patient representatives as full contributors with voting rights in policy decisions that affect practice and patient care. Sixth, issue an urgent call to societies and national funding bodies to recognize and fund the current gap in advanced practice provider research and policy with participatory research.

Advanced practice providers are not merely stopgaps but highly skilled professionals who contribute to safe, effective, and compassionate patient care. We advocate for a balanced, evidence-based approach to evaluating their roles, embracing opportunities to enhance their training, support their integration into health care teams, and recognize their contributions as essential members of the health care workforce.

## Abbreviations

NP: nurse practitioner
PA: physician associate
